# Biophysical Modeling of Dopaminergic Denervation Landscapes in the Striatum Reveals New Therapeutic Strategy

**DOI:** 10.1523/ENEURO.0458-21.2022

**Published:** 2022-03-02

**Authors:** Mathias L. Heltberg, Hussein N. Awada, Alessandra Lucchetti, Mogens H. Jensen, Jakob K. Dreyer, Rune N. Rasmussen

**Affiliations:** 1 Laboratoire de Physique, École Normale Supérieure, 75231 Paris Cedex 05, France; 2 Niels Bohr Institute, University of Copenhagen, 2100 Copenhagen, Denmark; 3 Section of Surgical Pathophysiology, University Hospital Copenhagen, 2200 Copenhagen, Denmark; 4 Department of Anesthesiology, University Hospital Copenhagen, 2200 Copenhagen, Denmark; 5 Department of Neuroscience, University of Copenhagen, 2200 Copenhagen, Denmark; 6 Department of Bioinformatics, H Lundbeck A/S, 2500 Valby, Denmark; 7 Center for Translational Neuromedicine, University of Copenhagen, 2200 Copenhagen, Denmark

**Keywords:** biophysics, Parkinson’s disease

## Abstract

Parkinson’s disease (PD) results from a loss of dopaminergic neurons. What triggers the break-down of neuronal signaling, and how this might be compensated, is not understood. The age of onset, progression and symptoms vary between patients, and our understanding of the clinical variability remains incomplete. In this study, we investigate this, by characterizing the dopaminergic landscape in healthy and denervated striatum, using biophysical modeling. Based on currently proposed mechanisms, we model three distinct denervation patterns, and show how this affect the dopaminergic network. Depending on the denervation pattern, we show how local and global differences arise in the activity of striatal neurons. Finally, we use the mathematical formalism to suggest a cellular strategy for maintaining normal dopamine (DA) signaling following neuronal denervation. This strategy is characterized by dual enhancement of both the release and uptake capacity of DA in the remaining neurons. Overall, our results derive a new conceptual framework for the impaired dopaminergic signaling related to PD and offers testable predictions for future research directions.

## Significance Statements

Parkinson’s disease (PD), caused by a loss of dopaminergic neurons, is the second most common neurodegenerative disorder worldwide. Clinically, the age of onset, disease progression, and symptoms are highly variable between patients. Despite this, an understanding of the underlying mechanisms causing this variability is still missing. We here use biophysical modeling and show that the spatial pattern of dopaminergic denervation profoundly affects the anatomy and signaling of the dopaminergic network. We further show that the pattern of denervation has functional consequences for the activity of the downstream projection neurons, critical for the direct and indirect pathways. Our findings are useful in understanding the clinical variability of PD and offers several experientially testable predictions.

## Introduction

Parkinson’s disease (PD) is a common neurodegenerative disorder, affecting 1% of people over the age of 60 worldwide (Hirtz et al., 2007). The disease is caused by a progressive loss of dopaminergic neurons in the substantia nigra pars compacta (SNc; Damier et al., 1999; Rodriguez-Oroz et al., 2009), and symptoms typically emerge when 60–80% of these neurons are lost (Fearnley and Lees, 1991; Lee et al., 2000). Notably, the age of onset, disease progression, response to treatment, and symptoms are highly variable between patients (Lewis et al., 2005; Greenland et al., 2019), pointing to a complex relationship between neuron loss and PD etiology that remains to be understood.

**Figure 1.**
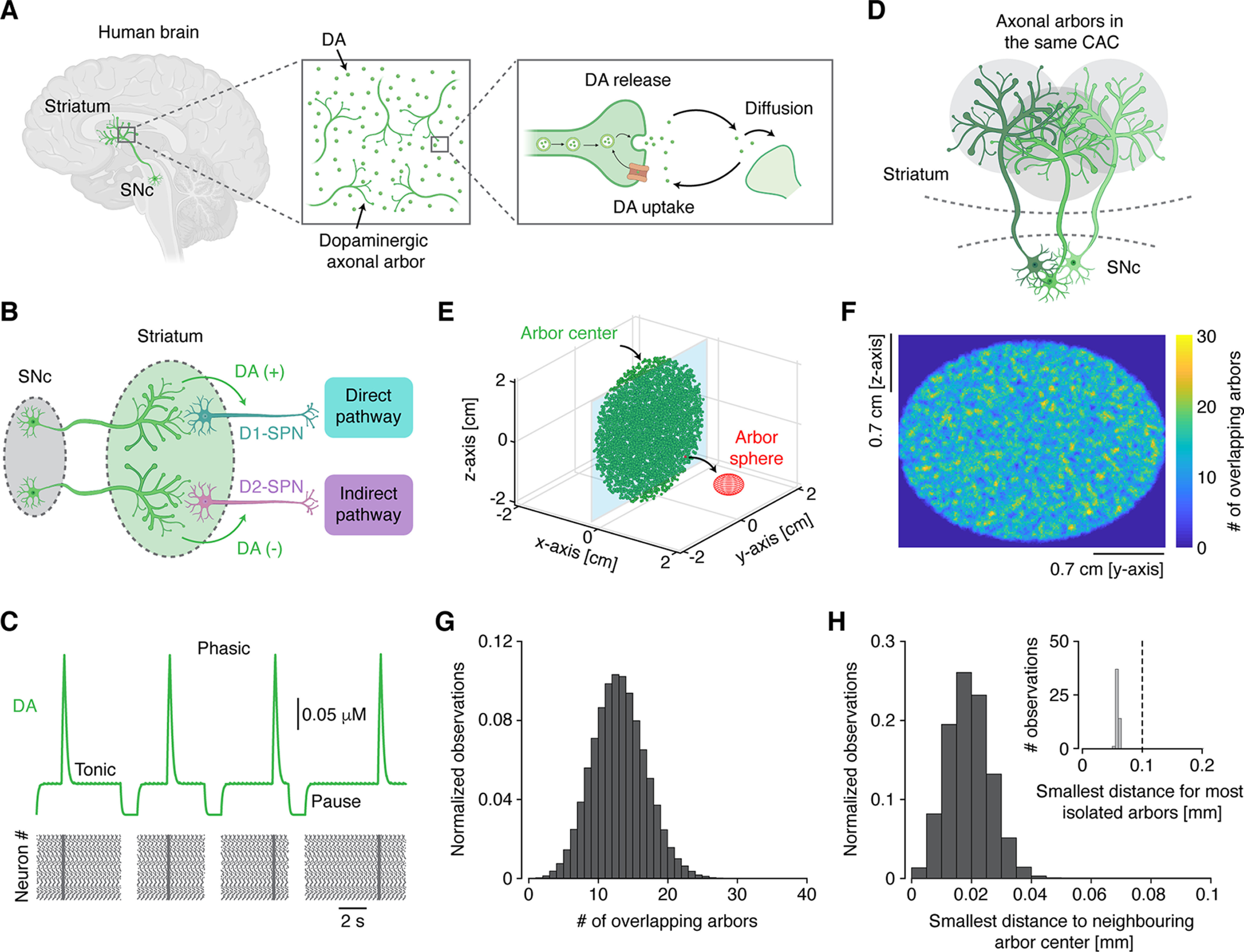
Functional and spatial characterization of DA signaling in the healthy human striatum. ***A***, Diagram of dopaminergic innervation and signaling in the human striatum. ***B***, Diagram of dopaminergic regulation of D1- and D2-SPNs, parts of the direct and indirect pathway, respectively. ***C***, Trace showing DA signaling and the underlying dopaminergic neuronal firing pattern. ***D***, Illustration of overlapping dopaminergic axonal arbors belonging to the same CAC. ***E***, Visualization of dopaminergic axonal arbors in the striatum; each arbor center is marked with a circle. For visibility, only 10% of arbors are shown. Red sphere shows the area subsumed by an arbor from one neuron. Notice that all arbors belong to the same CAC, represented by them all having the same color. ***F***, Heatmap of the distribution of overlapping arbors in the two-dimensional plane denoted in ***E***. ***G***, Distribution of the number of overlapping arbors for each individual arbor. ***H***, Distribution of the smallest distance to the nearest neighboring arbor center for each arbor. Inset, Smallest distance to nearest neighboring arbor center for the most isolated arbors found using Voronoi tessellation. DA, dopamine; SNc, substantia nigra pars compacta; D1, D1-class dopamine receptor; D2, D2-class dopamine receptor; SPN, spiny projection neuron; CACs, contiguous arbor classes. See also Extended Data 1 and Extended Data Figure 1-1.

Dopaminergic SNc neurons send projections to the dorsal striatum in the basal ganglia (Fig. 1*A*), an important area for motor function and executive control (Kreitzer and Malenka, 2008). These projections promote movement by modulating the excitability of GABAergic striatal spiny projection neurons (SPNs) by activating D1-class or D2-class dopamine (DA) receptors (Surmeier et al., 2007; Kreitzer, 2009). DA increases the excitability of D1 receptor-expressing SPNs (D1-SPNs) and decreases the excitability of D2 receptor-expressing SPNs (D2-SPNs; Kreitzer and Malenka, 2008; Lahiri and Bevan, 2020). D1- and D2-SPNs are critical components of two distinct pathways, traditionally thought to control movements in opposing ways: the direct pathway promotes desired movements while the indirect pathway suppresses unwanted movements (Kreitzer and Malenka, 2008; Kravitz et al., 2010; Cui et al., 2013; Freeze et al., 2013; Fig. 1*B*). In PD, dopaminergic neurons are progressively lost, leading to striatal DA depletion, abnormal SPN activity and movement deficits (Mazzoni et al., 2007; Rodriguez-Oroz et al., 2009; Kravitz et al., 2010). Despite the central role of failing DA signaling in PD etiology, little is known about the nature of striatal DA signaling before and during disease progression, posing a significant obstacle to the development of therapeutic strategies which maintain normal DA signaling in PD patients.

10.1523/ENEURO.0458-21.2022.ed1 Extended Data 1 Extended Data Equations. Mathematical models and algorithms. Folder containing a detailed description of all mathematical derivations and formulations, biophysical models, and algorithms employed for the study. Download Extended Data 1, ZIP file.

10.1523/ENEURO.0458-21.2022.f1-1 Extended Data Figure 1-1 Model parameters. Table summarizing the biophysical parameter values used for the respective computational models. Download Figure 1-1, TIF file.

Efforts focused on understanding the molecular cascades underlying PD neurodegeneration (Michel et al., 2016) have proposed different mechanisms, including the prion-hypothesis (Prusiner, 2012; Chu and Kordower, 2015) and oxidative stress (Jenner, 2003; Sulzer, 2007). However, little attention has been given to investigating the spatial and temporal patterns of dopaminergic neuron loss. Clinical imaging techniques, measuring DA transporter densities, provide a correlate of dopaminergic innervation (Wang et al., 2012; Ba and Martin, 2015) but cannot resolve the fine-scale organization of neurons at cellular resolution. In animal models, neuronal firing and DA signals can be recorded invasively (Patriarchi et al., 2018; Lippert et al., 2019) and correlated with dopaminergic neuron density postmortem. In addition to the challenge of being limited to a highly localized area, this approach lacks the temporal scale needed to track slow changes in neuron density and DA signaling.

Here, we developed a series of biophysical models to study how signals are lost by the denervation of dopaminergic neurons. Our results support a conceptual framework where the clinical manifestations of PD are rooted in the distinct denervation patterns and, importantly, provide theoretical predictions to be experimentally tested. Specifically, our work predicts that (1) variability in PD progression and symptoms stems from different spatiotemporal striatal denervation patterns caused by distinct cellular disease mechanisms, and (2) a dual cellular strategy, enhancing both release and uptake capacity of dopamine in remaining neurons, can counteract striatal signaling disruption caused by dopaminergic denervation.

## Materials and Methods

A detailed description of all mathematical derivations and formulations, biophysical models, and algorithms are included in the Extended Data 1. In brief, this work uses numerical and analytical mathematical methods to theoretically investigate and characterize the dopaminergic innervation of the human striatum, and the networks that break down following denervation. In the first part of the paper, we use mean-field theory to derive a differential equation describing dopamine (DA) signaling in a mesoscopic region of the striatum. By inducing three distinct periods of dopaminergic neuron firing, we solve this numerically. Following this, we introduce individual axonal arbors in the striatum, using the random generator applied in MATLAB. To characterize the organization and spatial coverage of arbors we analyze whether innervation was coherent within the striatum. Inspired by the mathematical analysis of communication classes, we quantify the number of what we termed contiguous arbor classes (CACs) and estimate the unoccupied space by placing 10,000 random points in the striatum. Next, we repeat these measures for the three denervation mechanisms: random denervation (RD), prion-like denervation (PLD), and stress-induced denervation (SID; described in further detail below). We then introduce a differential equation describing cAMP following DA stimulation, which we solve numerically. With this, we record the maximal cAMP value for both D1- and D2-SPNs. We next implement a Hodgkin–Huxley-inspired neuronal model and simulated this in Python using the Numba package. Using this, we measure the number of elicited action potentials in a short temporal window, in which the D1- and D2-SPNs were stimulated with the corresponding maximal cAMP level. This was done for 100 neurons at several different levels of denervation. Finally, we use the average number of action potentials recorded in this window as input for 10,000 randomly positioned points in the striatum, dependent on the denervation model and levels. Using these numbers, we calculate a spatial average and SD of the maximal firing rates of D1- and D2-SPNs. In this work, we did not employ statistical significance testing to compare conditions since all results were derived from analytical simulations and thus the major source of uncertainty is inherent to the chosen parameter values rather than variance across simulation iterations. All code is made publicly available on a GitHub repository: https://github.com/Mathiasheltberg/Theoretical_Denervation_ParkinsonsModel.

## Results

### Functional and spatial characterization of DA signaling in the healthy striatum

We began our investigation by modeling DA signaling in the fully innervated human striatum, specifically the putamen, which we defined as the healthy state (Dreyer et al., 2010; Dreyer, 2014). We simulated the firing of dopaminergic SNc neurons and described the extracellular DA concentration. For this, we employed a model describing DA in a subvolume of 10^3^ mm^3^ (see Extended Data 1). Given the estimated density of ∼0.1 dopaminergic axonal terminals per mm^3^ in the healthy striatum (Doucet et al., 1986; Dreyer et al., 2010; Dreyer, 2014), this volume contains on average 100 terminals, each of which was treated as an individual element. This is a reasonable approximation since each time a neuron fires, only a fraction of its terminals release transmitter. Estimates of the vesicular release probability of dopaminergic terminals are thus within the range of 6–20% (Dreyer, 2014; Pereira et al., 2016), and only ∼30% of the terminals may contain the molecular machinery for exocytosis (Liu et al., 2018). Based on this, we employed a deterministic mean-field model that approximates DA inside the *i*th subvolume as:

$$\frac{dM_{i}}{dt}=\text{Δ}\nu N_{i}-V_{M}N_{i}\frac{M_{i}}{K_{M}+M_{i}}+(D\nabla^{2}M_{i}-\epsilon M_{i}).$$

Here, *M* is the DA concentration, *Δ* is the amount of DA released by a terminal, *ν* is the neuronal firing frequency, *V_M_* is the DA uptake per terminal, and *N* is the number of terminals within the subvolume. DA remains active in the extracellular space until it is removed by either transporters or degraded enzymatically (Fig. 1*A*), so we modeled transporter-mediated DA uptake after the Michaelis-Menten uptake equation. We also included a simple degradation term (ϵ), and a term to account for the diffusion between neighboring subvolumes (*D*). These are, however, so small that they can be neglected if a region has dopaminergic innervation and will only be considered for regions deprived of terminals, and hence they are placed in parentheses. As shown in previous studies, we included that each dopaminergic neuron can express one of three firing patterns: pauses, tonic, or phasic (Grace and Bunney, 1984a, b). Thus, in the model, the firing frequency could take one of three values: 0 Hz (pauses), 4 Hz (for tonic), or 20 Hz (for phasic). All employed parameter values (Extended Data Fig. 1-1) were adopted from previous theoretical work which used experimentally determined measurements to constrain their model (Dreyer et al., 2010; see their Table 1). From this, we obtained DA time courses that clearly reflected the underlying neuronal firing patterns, exhibiting periods of tonic and phasic DA signaling, and pauses where DA is cleared from the extracellular space (Fig. 1*C*).

We next characterized the dopaminergic innervation of the striatum at a mesoscale. To mimic the shape of the putamen in the human striatum, we modeled it as an ellipsoid. Dopaminergic innervation was constructed by filling the volume with axonal arbors from 10^5^ SNc neurons, based on estimates from human SNc (Hardman et al., 2002) and the fact that these neurons have wide-spread projection targets (Poulin et al., 2018). Each neuron contributed with a spherical arbor with a radius of 0.5 mm (which is our best estimate based on the existing data), wherein the density of terminals was constant (Doucet et al., 1986; Matsuda et al., 2009). To characterize the organization and spatial coverage of arbors within the striatum, we analyzed whether innervation was coherent within the striatum. This can be quantified by the number of what we termed contiguous arbor classes (CACs), inspired by the mathematical analysis of communication classes. We assumed that dopaminergic neurons belonged to the same CAC if their arbors considerably overlapped (their arbor centers <0.5 mm apart; Fig. 1*D*). Hence, if the number of CACs is low, it suggests a high degree of spatial coverage and cohesion, and vice versa. For classifying neurons into CACs, we used a Markov chain-inspired algorithm (see Extended Data 1). We found that all neurons in the healthy striatum belonged to the same CAC (Fig. 1*E*), suggesting a high degree of coverage (Fig. 1*F*). For each neuron, we also counted the number of overlapping arbors, and this metric followed a Poisson distribution (Fig. 1*G*).

From the equation above, it is evident that decreasing $N_{i}$ does not affect the steady state DA concentration considerably unless this value is approximately zero. From calculations on the diffusion equation (see Extended Data 1), we determined that each point within the striatum with a distance larger than 0.1 mm to its nearest neighboring arbor was defined as isolated. We therefore searched for spatially isolated areas where the innervation was sparse, since such areas would be more susceptible to impairments in DA signaling during denervation. Using Monte Carlo simulations, we approximated the distribution of smallest distances and used Voronoi tessellation to find the most isolated points (Fig. 1*H*; see Extended Data 1). This showed that no isolated areas existed in the fully innervated striatum of our model. This result is dependent on the size of the arbors as well as their numbers in the healthy striatum, and we thus tested different values of both and found the system to be quite robust.

These results demonstrate that the modelled dopaminergic arbors comprise a network that densely covers the striatum, where no isolated areas exist.

### Different denervation patterns break down the dopaminergic network with distinct evolutions

In biology, structure often informs function. We therefore probed the spatial landscape of dopaminergic arbors in the denervating model striatum. The molecular pathways involved in the loss of dopaminergic neurons are beyond the scope of this study. Instead, we sought to characterize the organization of the remaining innervation arising from distinct models of progressive neuron loss.

**Figure 2.**
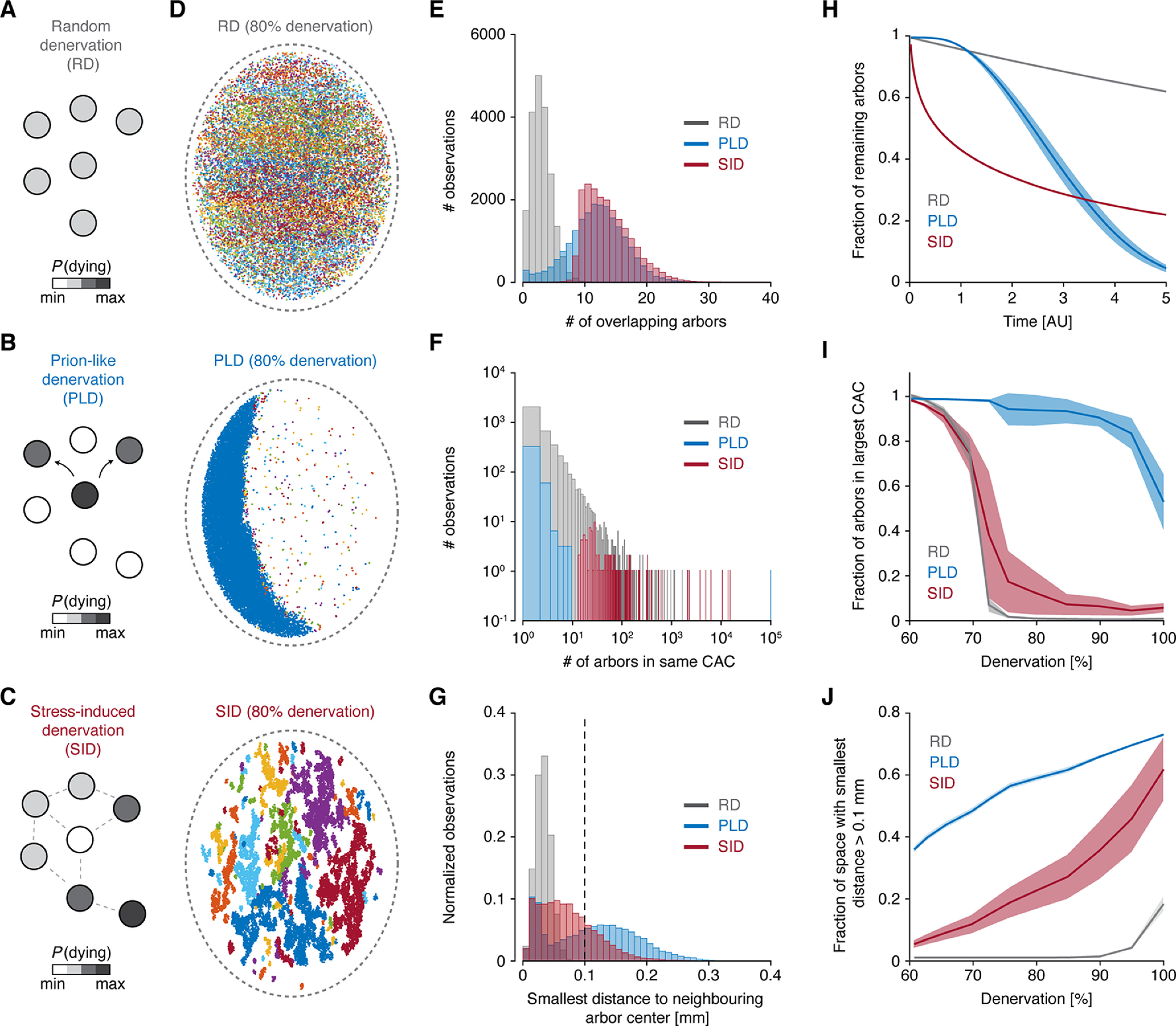
Different denervation patterns break down the dopaminergic network with distinct evolutions. ***A–C***, Diagrams of network mechanism for RD, PLD, and SID. The color of each dopaminergic neuron (circle) corresponds to probability of death. In ***C***, dotted lines denote overlap of arbors. ***D***, Visualization of the dopaminergic axonal arbor network following RD, PLD, and SID. Colors correspond to separate CACs. ***E***, Distributions of the number of overlapping arbors for each individual arbor. ***F***, Distributions of the number of arbors in each CAC. ***G***, Distributions of the smallest distance to the nearest neighboring arbor center for each arbor. Dotted line denotes threshold for classifying isolated areas. In ***E–G***, denervation is 80%. ***H***, Fraction of remaining arbors as a function of time. ***I***, Fraction of arbors belonging to the largest CAC as a function of denervation. ***J***, Fraction of striatal space with smallest distance to nearest arbor larger than 0.1 mm (isolated area) as a function of denervation. In ***H–J***, full line is mean, and shading is SD. RD, random denervation; PLD, prion-like denervation; SID, stress-induced denervation; CACs, contiguous arbor classes; AU, arbitrary unit. See also Extended Data Figure 2-1.

To describe denervation, we assumed that all neurons have a rate of dying. All models were simulated using the Gillespie algorithm, and neurons were removed according to their rate. We first modelled the denervation to be independent of the spatial position of a neuron, meaning that all neurons had the same rate of dying. This we termed random denervation (RD; Fig. 2*A*). The second model, prion-like denervation (PLD; Fig. 2*B*), one neuron was initially infected. All infected neurons then had a rate to infect others and dying, while all uninfected neurons had zero rate of dying. Based on the rates, one infected neuron was chosen to infect two neighboring neurons before being removed from the network. This algorithm was based on the proposed mechanisms where protein aggregates spread between neurons and cause their degeneration (Prusiner, 2012; Chu and Kordower, 2015; Surmeier et al., 2017). In the third model, stress-induced denervation (SID), each neuron has a rate of dying calculated as a sigmoidal function of its number of neighbors. Thus, neurons with few overlapping arbors have a higher risk of dying compared with neurons with many overlapping arbors. This algorithm was based on the proposed mechanism where the remaining neurons may upregulate their DA synthesis and firing activity to maintain DA signaling. However, these neurons may already be close to their maximal metabolic capacity (Bolam and Pissadaki, 2012), and increased activity could trigger stress-induced degeneration (Jenner, 2003; Sulzer, 2007). We want to emphasize, that we do not consider these three models as mutually exclusive nor the “ground truth” mechanisms for the process of dopaminergic denervation. However, they each represent a simple algorithm for studying the self-organization of these complex phenomena and has the potential to give important insight in the different denervation structures.

10.1523/ENEURO.0458-21.2022.f2-1 Extended Data Figure 2-1 Spatial denervation evolutions are robust to changes in key parameters. ***A***, Effects of varying the volume of axonal arbors (radius: 0.45, 0.5, or 0.55 mm) uniformly across the arbor population in the three denervation models. Upper, Fraction of arbors belonging to the largest CAC as a function of denervation. Lower, Fraction of striatal space with smallest distance to nearest arbor larger than 0.1 mm (isolated area) as a function of denervation. ***B***, Same as in ***A***, but with the volume of arbors following a δ function $\left(V_{n}=\delta \left(V_{n}-V_{0}\right)\right)$ versus heterogeneous distribution so the volume follows a normal distribution $\left(V_{n}=\frac{V_{0}}{10}\times N\left(0,1\right)\right)$. For both cases, $V_{0}=\frac{4}{3}\pi r_{0}$ where $r_{0}$ is the standard radius $\left(r_{0}=0.5mm\right)$. ***C***, Same as in ***A***, but for different numbers of dopaminergic neurons in the healthy state (80,000, 100,000, or 130,000 neurons). In ***A–C***, full line is the mean, and shading is the SD. RD, random denervation; PLD, prion-like denervation; SID, stress-induced denervation; CAC, contiguous arbor class. Download Figure 2-1, TIF file.

Using simulations, we observed how the three models resulted in distinct spatial landscapes, each characterized by a unique dopaminergic network breakdown (Fig. 2*D–G*). For RD, the remaining arbors covered the entire striatal space but no longer belonged to the same CAC. In contrast, for PLD, large fractions of the striatum were deprived of arbors and instead dominated by one or two subregions with seemingly normal innervation. For SID, arbors were concentrated in small, isolated subregions, each forming its own CAC. We quantified these observations by the distribution of the number of overlapping arbors (Fig. 2*E*). For PLD, a notable fraction of arbors had very low numbers of overlapping arbors, while a larger fraction had numbers like those in the healthy striatum. In SID, only arbors with many overlapping neighbors remained. Importantly, a commonality of all models was that the dopaminergic network broke down into multiple CACs, but in distinct patterns (Fig. 2*F*): RD had only small classes remaining, PLD contained many small but also one dominating class, whereas SID contained many classes containing 100 or more arbors. We also assessed the emergence of isolated areas (Fig. 2*G*). For RD, no isolated areas existed. In contrast, for both PLD and SID, the striatum contained numerous isolated areas, deprived of arbors.

Next, we followed spatial characteristics as a function of denervation. First, we determined the percentage of remaining arbors as a function of time (Fig. 2*H*). For RD, this followed an exponential decay with a relatively slow temporal progression. Interestingly, for PLD, the curve followed a convex function, suggesting that neuron loss accelerated with time, whereas the curve for SID followed a concave function, indicating that denervation in this scheme started fast, but then slowed with time. These results have predictive strength and can be mathematically described by stretched exponentials of the form: $N(t)\propto e^{-bt^{c}}$, with *b* being the decay rate and *c *=* *1 for RD, *c *>* *1 for PLD and *c *<* *1 for SID. We next characterized the breakdown of the spatial network, by calculating the fraction of arbors in the largest CAC (Fig. 2*I*). PLD kept one dominating class until the final stage of denervation, while RD and SID were characterized by a tipping point, at which the network dramatically transitioned from fully coherent to segregated into multiple classes. Notably, this transition occurred around 75% denervation, which often correlates with the onset of symptoms in PD patients (Bernheimer et al., 1973; Fearnley and Lees, 1991; Ma et al., 1997; Lee et al., 2000). Finally, we probed the emergence of isolated areas, practically devoid of DA signaling. At 75% denervation, isolated areas comprised ∼50% and 20% of the striatum in the PLD and SID models, respectively (Fig. 2*J*). At the same denervation level, no isolated areas existed for RD, but these emerged at severe denervation.

Overall, we found notable spatial and temporal differences between distinct models of dopaminergic denervation. These differences between models were remarkably robust to changes in key parameters, that is, axonal arbor volume and numbers of dopaminergic neurons in the healthy state (Extended Data Fig. 2-1).

### Dopaminergic denervation affects cAMP production and the activity of striatal SPNs

**Figure 3.**
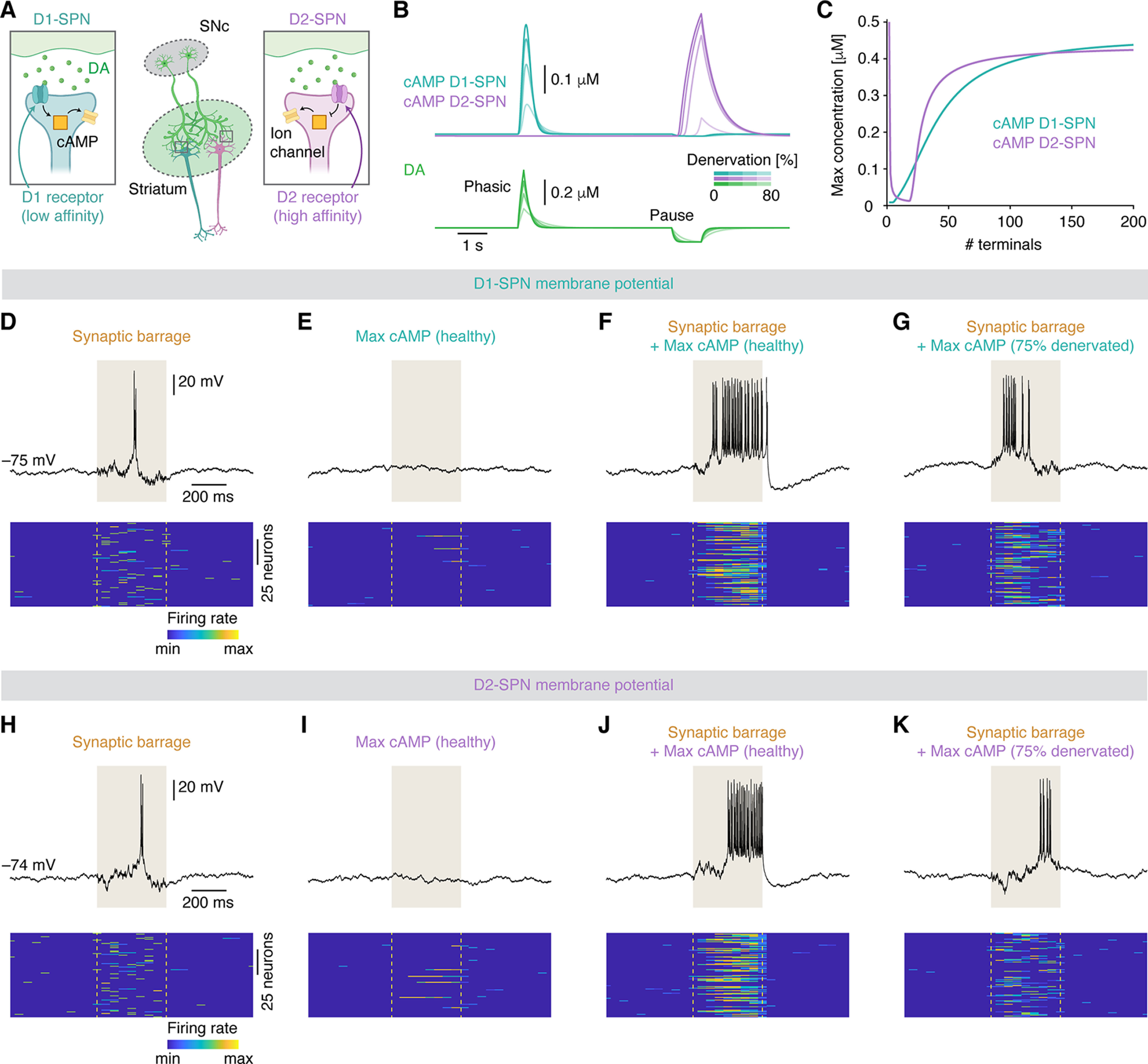
Dopaminergic denervation affects cAMP signaling and excitability of striatal SPNs. ***A***, Diagram of how DA stimulates and inhibits the production of cAMP in D1- and D2-SPNs, respectively. ***B***, Traces showing cAMP in D1- and D2-SPNs as a function of DA signaling. ***C***, Maximal cAMP concentration in D1- and D2-SPNs during dopaminergic phasic firing and firing pauses, respectively, as a function of the number of dopaminergic terminals. ***D–K***, Membrane potential dynamics of D1- and D2-SPNs in response to synaptic barrages (***D***, ***H***), cAMP stimulation (***E***, ***I***), synaptic barrages in combination with cAMP stimulation in the healthy state (***F***, ***J***), or synaptic barrages in combination with cAMP stimulation in the 75% denervated state (***G***, ***K***). Raster plots show the firing rate across time for 100 simulated neurons in each condition. DA, dopamine; D1, D1-class dopamine receptor; D2, D2-class dopamine receptor; SPN, spiny projection neuron; SNc, substantia nigra pars compacta. See also Extended Data Figure 3-1.

The excitability of SPNs is, along with several other factors, strongly regulated by DA (Surmeier et al., 2007; Kreitzer and Malenka, 2008; Kreitzer, 2009; Lahiri and Bevan, 2020). Thus, we next asked how dopaminergic denervation affects the activity of individual SPNs. Previous work has shown that D1 and D2 receptors have low and high DA affinity, respectively (Richfield et al., 1989; Fig. 3*A*). DA regulation of SPN excitability is mediated by the signaling molecule cAMP. D1 and D2 receptor activation increases and decreases the production of cAMP, respectively, and cAMP in turn regulates SPN ion channels (Surmeier et al., 2007; Kreitzer and Malenka, 2008; Kreitzer, 2009). Inspired by previous work (Dreyer et al., 2010), we described the intracellular cAMP concentration in D1- and D2-SPNs as:

$$\frac{dcAMP_{\text{D}1}}{dt}=\alpha +\lambda_{1}\frac{DA^{h}}{DA^{h}+\kappa_{1}^{h}}-\delta_{1}cAMP_{\text{D}1}$$

$$\frac{dcAMP_{\text{D}2}}{dt}=\alpha +\lambda_{2}\frac{\kappa_{2}^{h}}{DA^{h}+\kappa_{2}^{h}}-\delta_{2}cAMP_{\text{D}2}.$$

10.1523/ENEURO.0458-21.2022.f3-1 Extended Data Figure 3-1 Distinct denervation patterns differentially affect local and global striatal SPN firing activity in the Izhikevich model. ***A***, Membrane potential of D1-SPN (left) and D2-SPN (right) in the healthy and 75% denervated striatum in response to DA signaling, modelled using the Izhikevich model. ***B***, ***C***, Maximal firing activity of D1- and D2-SPNs across space in the healthy and 75% denervated striatum for the three denervation patterns: RD, PLD, and SID. ***D***, ***E***, Spatial mean and SD of maximum firing activity in D1- and D2-SPNs as a function of denervation. SPN, spiny projection neuron; D1, D1-class dopamine receptor; D2, D2-class dopamine receptor; DA, dopamine. Download Figure 3-1, TIF file.

Here, α is the steady state production of cAMP, and δ is its spontaneous decay. In addition, receptor-dependent cAMP production was implemented: cAMP in D1- and D2-SPNs increased and decreased with DA stimulation, respectively. With increasing denervation, cAMP production during phasic firing became progressively lower in D1-SPNs, while in D2-SPNs it became progressively lower during firing pauses (Fig. 3*B*,*C*).

We next asked how these impairments in cAMP signaling may manifest in the activity of SPNs. For this, we used a previously published Hodgkin–Huxley-inspired model (Tatsuki et al., 2016; Rasmussen et al., 2017) to simulate the membrane potential (V_m_) of D1- and D2-SPNs. This model contains extrinsic and intrinsic ion channel conductances. The extrinsic conductances are NMDA, AMPA, and GABA_A_ ion channels. The intrinsic conductances are voltage-gated and persistent Na^+^ channels, voltage-gated Ca^2+^ channels (Ca_V_), and voltage-gated, leak, fast A-type, inwardly rectifying, slowly inactivating (K_SI_), and Ca^2+^-dependent (K_Ca_) K^+^ channels (Tatsuki et al., 2016; Rasmussen et al., 2017; see Extended Data 1). Using this model, we could closely mimic the V_m_ dynamics of SPNs. These neurons are characterized by their transitions between downstates and upstates (Wickens and Wilson, 1998; Sippy et al., 2015). In our model, on increased levels of synaptic barrages (implemented by increasing the stochastic noise of the V_m_), both D1- and D2-SPNs transitioned into a brief upstate in which multiple action potentials were fired (Fig. 3*D*,*H*).

To implement the effect of DA, via its regulation of cAMP, on the V_m_ dynamics of D1- and D2-SPNs, we targeted the high-threshold Ca_V_ (N-type and P-type), K_SI_, and K_Ca_ channels, which are negatively influenced by DA and cAMP signaling (Surmeier and Kitai, 1993; Nisenbaum et al., 1994; Surmeier et al., 1995, 2007; Shen et al., 2004; Lahiri and Bevan, 2020). Thus, for increasing cAMP levels, the conductance of these channels decreases and vice versa. We note that other channels may also be subject to dopaminergic modulation, such as persistent Na^+^ or NMDA channels, but to limit the parameter space, we here focus on the above mentioned Ca^2+^ and K^+^ channels. For stimulating D1- and D2-SPNs, we used the cAMP concentrations observed during dopaminergic phasic firing and firing pauses, respectively. This was motivated by the result that, in the healthy striatum, the maximal cAMP production in D1- and D2-SPNs was observed during these two phases respectively (Fig. 3*B*). In itself, cAMP stimulation was very rarely sufficient to evoke a transition from the downstate to an upstate in types of SPNs (Fig. 3*E*,*I*), supporting the notion of DA as a “modulator” rather than a “driver” (Surmeier et al., 2007; Kreitzer, 2009; Lahiri and Bevan, 2020). However, if DA and cAMP stimulation coincided with increased levels of synaptic barrages, this triggered a robust upstate that lasted longer and elicited more action potentials than with synaptic barrages alone (Fig. 3*F*,*J*); this demonstrates that cAMP powerfully regulates the excitability of D1- and D2-SPNs. As a result, in the denervated state, the activity of SPNs was notably affected (Fig. 3*G*,*K*): the duration of the upstate and the firing rate during the upstate were strongly diminished in both D1- and D2-SPNs. These findings were replicated using the simpler Izhikevich model (Extended Data Fig. 3-1), suggesting model-invariance.

Together, these data demonstrate that DA signaling, via its downstream effector cAMP, can regulate the firing activity of SPNs, and this regulation is impaired in the denervated state.

### Distinct denervation patterns differentially affect global SPN firing activity

**Figure 4.**
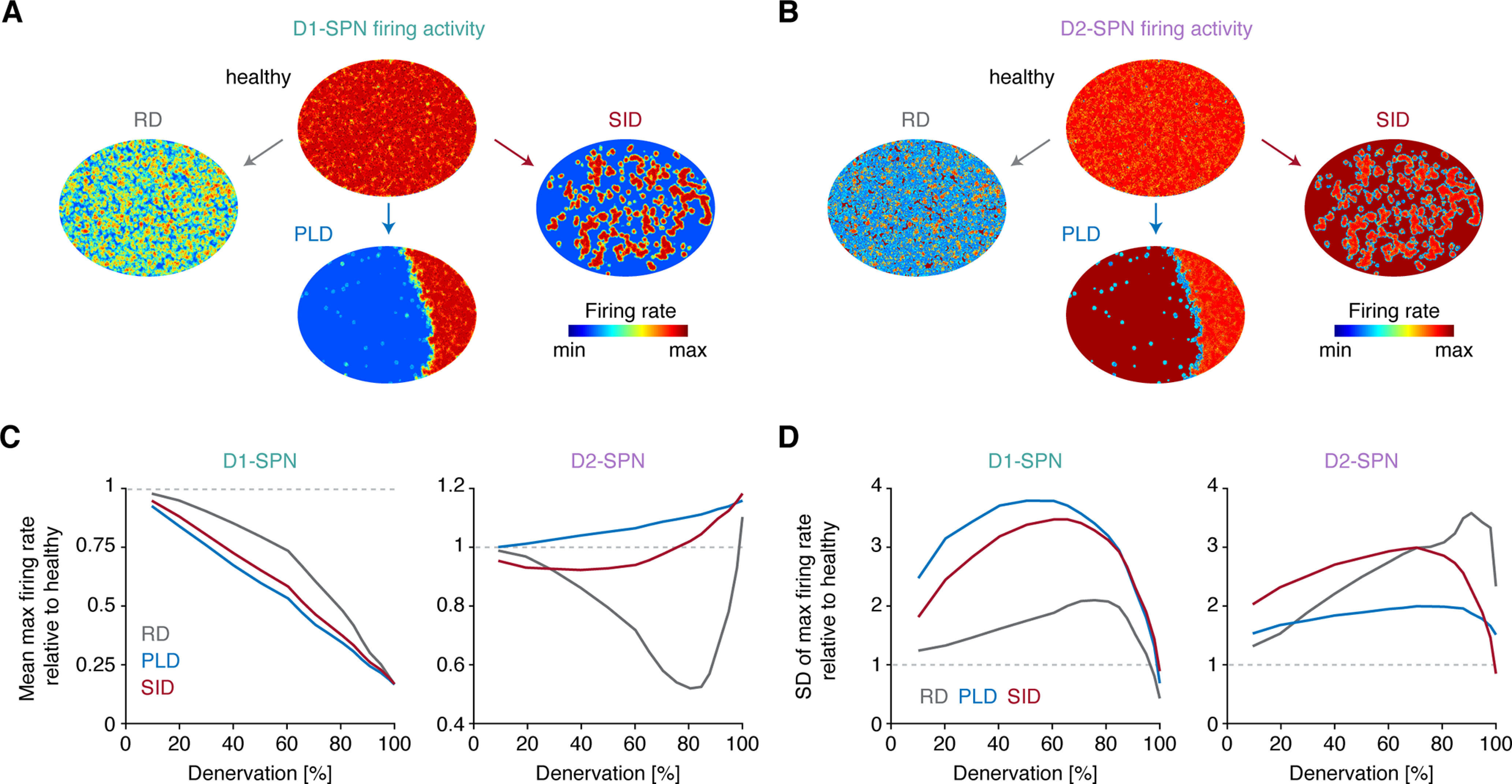
Distinct denervation patterns differentially affect global striatal SPN firing activity. ***A***, ***B***, Maximal firing activity of D1- and D2-SPNs across space in the healthy and 75% denervated striatum for the three denervation patterns. ***C***, ***D***, Spatial mean and SD of maximal firing activity in D1- and D2-SPNs as a function of denervation. RD, random denervation; PLD, prion-like denervation; SID, stress-induced denervation; D1, D1-class dopamine receptor; D2, D2-class dopamine receptor; SPN, spiny projection neuron.

Next, we sought to investigate how different denervation patterns affect the activity of D1- and D2-SPNs across striatal space. For this, we spatially mapped the maximal firing activity for both types of neurons; D1-SPN during phasic firing and D2-SPN during firing pauses using the Hodgkin–Huxley-inspired model (Fig. 4*A*,*B*). In RD, although almost all the subregions had relatively low DA levels compared with the healthy striatum, this was still sufficient to evoke intermediate D1-SPN firing rates across the extent of the striatum. In contrast, in both PLD and SID, D1-SPN firing was high only in the subregions with preserved DA innervation. Noticeably, PLD transformed the striatum into a strongly polarized activity map, whereas SID caused local heterogeneity. For D2-SPNs, the emergence of isolated areas, resulted in a very different outcome. Since the maximal DA concentration in isolated areas is zero (except for small diffusive fluctuations), D2-SPN firing rates were here very high, most profoundly expressed for PLD and SID. We note here that, under physiological conditions, D2-SPNs residing in regions deprived of DA signaling might adapt by downregulating their firing rates to maintain homeostasis. In this scenario, the results would likely be like those for D1-SPNs.

Finally, we characterized SPN activity in the three denervation models as a function of denervation. The mean D1-SPN firing rates decreased linearly as a function of denervation in all models (Fig. 4*C*). We also noted that the SD of D1-SPN firing was smaller in RD compared with both PLD and SID, indicating spatial homogeneity of firing levels (Fig. 4*D*). The effect on mean D2-SPN firing rates was different: firing increased notably for PLD and slightly for SID, as a function of denervation (Fig. 4*C*). In RD, the firing rates decreased until it reached a minimum around 80% denervation, whereafter it rapidly increased. This observation is explained by the occurrence of isolated areas, deprived of DA signaling, resulting in a dramatic increase in cAMP production in D2-SPNs (Fig. 3*C*), in turn resulting in a profound increase in excitability. Comparing the three models, the early progression of denervation (up to ∼60%) resulted in increased SD of SPN firing rates for all denervation patterns (Fig. 4*D*). This increase in activity variance across neurons may thus be a fingerprint of the denervating striatum. All the described results were fully replicated with the Izhikevich model (Extended Data Fig. 3-1).

Overall, these results show that the global firing activities of D1- and D2-SPNs are strongly affected by the specific spatial pattern of dopaminergic denervation.

### A dual presynaptic compensation strategy preserves DA signaling in the denervated striatum

**Figure 5.**
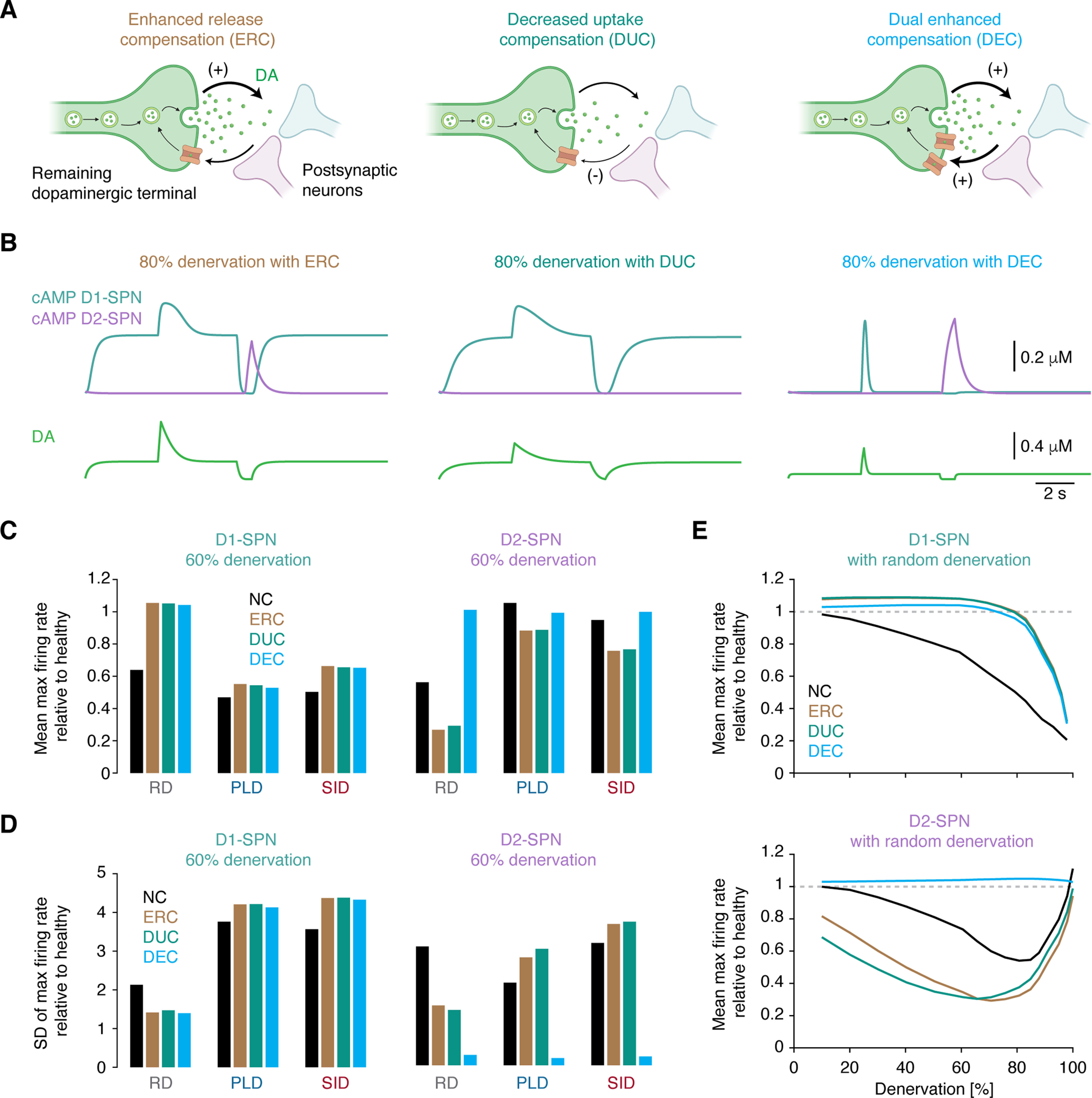
A dual presynaptic compensation strategy preserves DA signaling in the denervated striatum. ***A***, Diagrams of mechanisms of the ERC, DUC, and DEC models. ***B***, Traces showing cAMP in D1- and D2-SPNs as a function of DA signaling at 80% denervation in the compensation models. ***C***, ***D***, Spatial mean and SD of maximal firing activity in D1- and D2-SPNs as a function of denervation pattern and compensation model. ***E***, Spatial mean of maximal firing activity in D1- and D2-SPNs as a function of denervation and compensation model in the randomly denervated striatum. ERC, enhanced release compensation; DUC, decreased uptake compensation; DEC, dual enhanced compensation; NC, no compensation; DA, dopamine; D1, D1-class dopamine receptor; D2, D2-class dopamine receptor; SPN, spiny projection neuron; RD, random denervation; PLD, prion-like denervation; SID, stress-induced denervation. See also Extended Data Figures 5-1, 5-2.

Given that dopaminergic neurons loss may trigger compensatory mechanisms in the remaining neurons in an attempt to maintain normal DA signaling (Zigmond, 1997; Brotchie and Fitzer-Attas, 2009), we sought to probe the potency of such mechanisms, to attempt predicting ideal therapeutic strategies. We included three presynaptic compensatory mechanisms as perturbed parameter values (*V_M_* and *Δ*) in our original model for DA concentration dynamics. First, remaining dopaminergic terminals may increase their DA release capacity (Zigmond et al., 1990; Zigmond, 1997; Greenbaum et al., 2013). We refer to this as enhanced release compensation (ERC; Fig. 5*A*):

$${\text{Δ}}_{+}\mapsto \frac{{\text{Δ}}_{0}}{1-\delta }.$$

10.1523/ENEURO.0458-21.2022.f5-1 Extended Data Figure 5-1 Three distinct postsynaptic mechanisms fail to preserve DA signaling in the denervated striatum. ***A–C***, Diagrams of the modelled postsynaptic compensatory mechanisms: increased D2 expression (***A***), enhanced D1 and D2 sensitivity (***B***), and suppressed cAMP degradation in D1- and D2-SPNs mediated by, for example, a PDE inhibitor (PDE-I; ***C***). ***D–F***, Example traces showing cAMP in D1- and D2-SPNs as a function of DA signaling at 0% and 80% denervation in the different postsynaptic compensation models. DA, dopamine; D2, D2-class dopamine receptor; D1, D1-class dopamine receptor; SPN, spiny projection neuron; PDE, phosphodiesterase. Download Figure 5-1, TIF file.

10.1523/ENEURO.0458-21.2022.f5-2 Extended Data Figure 5-2 A dual presynaptic compensation strategy preserves SPN firing activity in the Izhikevich model in spite of severe denervation. ***A***, ***B***, Spatial mean and SD of maximum firing activity in D1- and D2-SPNs as a function of denervation pattern (RD, PLD, and SID) and compensation model (ERC, DUC, and DEC). ***C***, Spatial mean of maximum firing activity in D1- and D2-SPNs as a function of denervation and compensation model in the randomly denervated striatum. SPN, spiny projection neuron; D1, D1-class dopamine receptor; D2, D2-class dopamine receptor; RD, random denervation; PLD, prion-like denervation; SID, stress-induced denervation; ERC, enhanced release compensation; DUC, decreased uptake compensation; DEC, dual enhanced compensation. Download Figure 5-2, TIF file.

Here, we introduced the compensation parameter δ, a sigmoidal function going from zero to one as a function of the number of dopaminergic arbors covering a small volume. The parameter ${\text{Δ}}_{0}$ refers to the DA release in healthy subregions, whereas ${\text{Δ}}_{+}$ is the compensated release value. Second, DA transporters, expressed on terminals, may reduce their uptake capacity (Zigmond et al., 1990; Zigmond, 1997; Lee et al., 2000; Greenbaum et al., 2013). We refer to this as decreased uptake compensation (DUC; Fig. 5*A*):

$$V_{-}\mapsto V_{0}(1-\delta ).$$

As above, the parameter $V_{0}$ refers to the uptake value in healthy subregions, whereas $V_{-}$ is the compensated uptake strength. Finally, we suggest a mechanism where neurons compensate by enhancing both DA release and uptake capacity in the terminals. This idea stems from the equations for DA concentration dynamics, from where we can show mathematically that this combination recovers the original equations. Such a compensatory mechanism has not previously been suggested, and we refer to this as dual enhanced compensation (DEC; Fig. 5*A*); this is included in the model through changes in both the uptake and release parameters:

$${\text{Δ}}_{+}\mapsto \frac{{\text{Δ}}_{0}}{1-\delta }\text{and}V_{+}=\frac{V_{0}}{1-\delta }.$$

Here, all parameters are defined as above.

With these implementations, we simulated DA signaling and the corresponding cAMP production in D1- and D2-SPNs with 80% denervation. As shown above, in the absence of compensation, DA release during tonic firing is unaffected, but notably affected during phasic firing and firing pauses (Fig. 3*B*). Here, during tonic firing, the DA concentration notably increased for ERC and DUC models; during phasic firing, DA was increased for ERC, and during firing pauses, DA removal was incomplete for DUC (Fig. 5*B*). Importantly, the DEC model preserved DA levels during both tonic and phasic firing at comparable levels to in the healthy state, while still allowing complete DA removal during firing pauses (Fig. 5*B*). We also tested several postsynaptic compensatory mechanisms previously proposed in the literature, including increased D2 receptor expression (Guttman and Seeman, 1985), enhanced D1 and D2 receptor sensitivity (Lee et al., 1978), and suppressed cAMP degradation in D1- and D2-SPNs (Niccolini et al., 2015). These mechanisms were inadequate to restore DA and cAMP signals in SPNs (Extended Data Fig. 5-1), and we therefore did not explore these further.

Next, we asked whether any of the compensation mechanisms were able to counteract the impairments in SPN firing activity in the denervated state (Figs. 3, 4). For this we calculated the spatial mean and SD of the maximal D1- and D2-SPN firing rates in the three denervation models and combined these with the presynaptic compensation mechanisms (Fig. 5*C*). Interestingly, in the scenario of RD, only the DEC model preserved the mean level of D1- and D2-SPN firing rates. In contrast, for PLD and SID, none of the three compensation models were able to counteract the decrease in D1-SPN firing with denervation, while all models performed relatively well for D2-SPN firing activity. For the SD of the D1-SPN firing rates, we note that in the RD scenario, all compensation models, as well as the noncompensation state, maintained this measure near the healthy level (Fig. 5*D*). In contrast, for PLD and SID, the SD was notably increased for all compensation models, and curiously, the noncompensated state was most similar to the healthy state. For the SD of the D2-SPN firing activity, none of the compensation models truly maintained this measure close to the healthy level, regardless of the denervation pattern (Fig. 5*D*). It is here worth noting that the DEC model, across all denervation patterns, maintained the SD of D2-SPN firing activity at a very low level. This is because, in regions with low dopaminergic coverage, DA signaling from remaining terminals in the DEC model can compensate, restoring coherent neuronal activity. The low SD in firing activity across neurons means that all striatal subregions are capable of generating a very similar firing response on dopaminergic stimulation. Overall, we conclude that the DEC model, in combination with the RD pattern, best preserved the SPN firing activity. In the final set of simulations, we thus explored this for all levels of denervation (Fig. 5*E*). For the DEC model, the firing rates of both D1- and D2-SPNs remained remarkably close to the healthy state, despite reaching severe denervation. In contrast, for the ERC and DUC models, even at intermediate denervation, SPN firing differed from the healthy state. Therefore, the ERC and DUC mechanisms do not seem ideal as therapeutic strategies. These findings were replicated with the Izhikevich model (Extended Data Fig. 5-2).

Taken together, these results show that an ideal strategy to maintain normal SPN activity is to locally introduce a dual compensation mechanism, increasing both DA release and uptake capacity, and to globally minimize the dopaminergic arbor density differences, or at least avoid the emergence of isolated areas.

## Discussion

In this work, we used biophysical and mathematical modeling to investigate the spatial and functional landscape of dopaminergic signaling in the healthy and parkinsonian striatum. First, we showed that the spatial pattern of dopaminergic denervation profoundly affects the structural and temporal breakdown of the dopaminergic network in the striatum. Second, we derived how the local and global activity of D1- and D2-SPNs were differentially affected as a function of the spatial dopaminergic denervation pattern. Third, we identified that a combination of enhanced DA release and uptake capacity, presents a feasible strategy for maintaining the normal striatal DA signaling, when neurons are progressively lost.

### Clinical variability may be mediated by different dopaminergic denervation patterns

PD symptoms often present when 60–80% of dopaminergic neurons are lost (Fearnley and Lees, 1991; Ma et al., 1997). Still, the age of onset, disease progression and symptoms can vary notably between patients (Lewis et al., 2005; Greenland et al., 2019). We believe that the spatial pattern of denervation might play a role in this clinical variability. Our model shows that the dopaminergic denervation pattern may lead to different DA signaling landscapes. After an initial slow denervation rate, the loss of neurons accelerated with time in the PLD model. In contrast, in the SID model, denervation slowed with time after an initial rapid loss of neurons. The breakdown of CACs in the PLD and SID models correlates well with the clinical progression pattern seen in the early and middle stages of PD (Eckert et al., 2007; Liu et al., 2015). In patients with RD, disease progression may be slow, whereas in patients with PLD, the progression may accelerate rapidly. Hence, if the total density of striatal dopaminergic terminals could be measured as a function of time in the early stages of the disease, our model would predict the denervation pattern causing PD in individual patients. Assuming that different denervation landscapes stem from distinct molecular mechanisms within the dopaminergic neurons, a novel experimental technique called mass synaptometry (Gajera et al., 2019, 2021) could be exploited to obtain information about the molecular profile of the synapses in the remaining neurons; allowing the possibility to cross correlate the two predictions and ideally validating or rejecting our model. A possible limitation of this approach is that mass synaptometry was performed *in vitro* from postmortem brain samples (Gajera et al., 2019). Thus, if samples are obtained from PD patients after their death only, this would not necessarily reveal the molecular mechanisms in the early phases of the disease, so ideally, the optimal approach would be to obtain samples from patients while alive and as early as possible. This could potentially be obtained during neurosurgery for implanting deep brain stimulation electrodes. Furthermore, it may be feasible to, at least in part, predict the disease progression time course, and from that determine the ideal therapeutic strategy for the individual patient. We therefore propose that future clinical experiments aim to measure the density of dopaminergic terminals in the striatum of PD patients over time and their molecular characterization, using for example single-photon emission computed tomography (Wang et al., 2012; Ba and Martin, 2015) and mass synaptometry, and to correlate this to disease progression. Combining the results from such experiments with biophysical modeling would elucidate the molecular and network mechanisms causing PD and disease variability. We also found that the absolute time course of dopaminergic denervation was remarkably distinct between the different denervation patterns (Fig. 2), and this observation could potentially aid clinicians in determining the differential diagnosis of parkinsonism. Clinical imaging of early-stage PD patients has shown that structural innervation differences in the striatum, albeit embracing a notably larger striatal area than our results, relates to different PD-related diseases. For example, large-scale asymmetry in striatal dopaminergic innervation associates with idiopathic parkinsonism (Kim et al., 2002; Ziebell et al., 2012), while large-scale symmetric denervation associates with atypical parkinsonian syndromes such as supranuclear palsy (Varrone et al., 2001; Knudsen et al., 2004; Filippi et al., 2006; Ziebell et al., 2012). This difference might be, at least in part, explained by the three denervation patterns described in our work. Thus, the denervation curves from our model, in combination with high-resolution imaging and the mass synaptometry technique, might portent a valuable tool for distinguishing between different forms of parkinsonism in individual patients.

### Distinct denervation patterns may differentially affect the direct and indirect pathway

When dopaminergic neurons were lost, the burst firing during upstates of D1- and D2-SPNs was severely impaired (Fig. 3). Given that D1- and D2-SPNs are critical components of the direct and the indirect pathways, respectively, it is plausible that these two pathways would be affected as a result. The reduction of firing in D1-SPNs during phasic dopaminergic firing may complicate the initiation of voluntary movements, while the impaired firing in D2-SPNs during dopaminergic firing pauses may facilitate unwanted, involuntary movements. Given that, in the denervated striatum, DA signaling and SPN activity varied across space depending on the denervation pattern, we expect that different subregions of the striatum will have normal and abnormal activity of the direct and indirect pathways, depending on the denervation pattern. This may contribute to why disease symptoms can vary notably between PD patients. We mention this with the caveat that our simulations of SPN activity have their limitations. For example, DA is not the only modulator of SPN activity; also, local GABAergic and cholinergic interneurons regulate the activity of SPNs (Kreitzer, 2009; Burke et al., 2017), and our simulations do not account for that. Furthermore, we here investigated the acute effects of dopaminergic denervation and our results do thus not take into account the long-term changes in glutamatergic synaptic activity that may develop in the striatum as a result of denervation (Surmeier et al., 2007; Kreitzer and Malenka, 2008), nor did we explore possible changes in dopaminergic autoregulation (Ford, 2014). Future work should aim to investigate the interplay between different dopaminergic denervation patterns and these other mechanisms regulating SPN activity, for example in a more comprehensive basal ganglia network model, as recently developed (Hjorth et al., 2020).

### A dual cellular strategy could promote normal DA signaling

The most common pharmacological treatment for PD is to administer levodopa, with the goal of increasing DA levels within the brain (LeWitt, 2008; Hauser, 2009; Salat and Tolosa, 2013). However, not all patients respond well to this treatment, and some experience side effects with long-term treatment (LeWitt, 2008; Salat and Tolosa, 2013). During the early stages of PD, the dopaminergic neuron loss is believed to be counterbalanced by endogenous compensatory mechanisms (Zigmond, 1997; Brotchie and Fitzer-Attas, 2009). Knowledge of such mechanisms could reveal potential targets for novel therapeutic strategies (Brotchie and Fitzer-Attas, 2009). In our work, we found that DA signaling cannot be fully characterized merely by its tonic level, since the correct occurrence of peaks during phasic firing and the complete removal of DA during firing pauses likely plays important roles in proper neuronal signaling. Therefore, when evaluating the therapeutic potential of a cellular target, it is important to assess its effects on the full DA signaling spectrum. Through our investigation we found that the optimal approach to minimize the dopaminergic signaling effects of denervation, is to upregulate the released DA in combination with enhancing its uptake. While the first is typically achieved in patients by administering levodopa (Hauser, 2009), the latter has not, to our knowledge, been attempted yet, and could in principle be achieved by enhancing the level of dopamine transporters in the neurons. Therefore, this theoretical work suggests a new therapeutical strategy, where levodopa is given in combination with enhancement of dopamine transporters to maintain the steady state level of DA while at the same time recovering the fast changes in DA concentration during the short transient intervals of bursts and pauses. In contrast to other mechanisms, this dual mechanism preserved the DA signaling spectrum, without increasing tonic DA levels. We note that clinically this would involve a combinatorial treatment with, for example, levodopa and a genetic approach for boosting the synthesis of DA transports, which we believe will be possible to test in the future. Thus, if some neurons remain, this mechanism can restore the DA signaling properties, emphasizing the great importance of avoiding areas completely devoid of DA terminals. We hypothesize that this dual strategy might postpone the onset of severe symptoms by upholding normal DA signaling and could potentially cause less side effects since baseline DA is maintained at a comparable level to that in the healthy striatum.

In conclusion, our work constitutes a new conceptual model for the impairments of dopaminergic signaling related to PD. In this, we developed a holistic framework linking the activity of individual neurons to the spatiotemporal dopaminergic signaling landscape, while providing a clear set of theoretical predictions and testable hypotheses. We regard our biophysical modeling as the first step toward further experimental investigations required to test our results in animal models and ultimately in PD patients. We hope that the present work will lay the groundwork for new research directions within both basic and clinical neurosciences, aimed at better understanding and treating PD.

## Synthesis

Reviewing Editor: Mikko Airavaara, Helsingin yliopisto Decisions are customarily a result of the Reviewing Editor and the peer reviewers coming together and discussing their recommendations until a consensus is reached. When revisions are invited, a fact-based synthesis statement explaining their decision and outlining what is needed to prepare a revision will be listed below. The following reviewer(s) agreed to reveal their identity: Andrii Domanskyi. Note: If this manuscript was transferred from JNeurosci and a decision was made to accept the manuscript without peer review, a brief statement to this effect will instead be what is listed below.

Manuscript entitled “Theoretical analysis of dopaminergic denervation landscapes in the striatum suggests new therapeutic strategies” by Heltberg et al is well and logically written with schematic images for ease of understanding. Manuscript is on very interesting topic. Authors dwells into changes in innervation or synaptic changes that is one of the structures that gets affected during initial stage of neurodegeneration far before onset of cellular degeneration or clinical symptoms. Authors specifically studies biophysical modelling and show that the spatial pattern of dopaminergic denervation profoundly affects the anatomy and signaling of the dopaminergic network. Although dopaminergic denervation landscapes in the striatum in Parkinson’s disease is well documented and established fact, Such findings are useful in understanding the clinical variability of Parkinson’s disease and will help in paving way for new approaches.

The work is purely theoretical and so the validation of model predictions in real biological in vitro an in vivo settings is, unfortunately, beyond the scope of this study. Nevertheless, as rightfully noted by the authors, it provides testable predictions for the future research in biological models.

To improve the manuscript and readability following minor edits/ clarification are suggested to include in manuscript along with discussion of latest literature.

1) Please state in simple words and clearly how this approach suggest “new” therapeutic strategy or advance bioassay for searching novel compounds as therapeutics. Please discuss this in discussion section ( also in connections with below points)

2) Authors specifically studies biophysical modelling; how does this fit and relates with synaptic molecular composition changes in Parkinson’s disease with recent new technology called mass synaptometry analysis of single synapses and its mass tag barcoding of multiples samples leading to removal of variability and comparing finer differences among the molecular signatures. Please discuss how molecular and biophysical changes may be complementary for more detailed information.

3) how throughput and effective is this new analysis method therapeutic strategies and how much differences does this method intend to capture. In other word what is co-efficient of variation among the readouts from same samples prepared and run multiple times and what is difference between the experimental groups expected.

4) Optional: Authour may want to revise title of the paper to better reflect their work. e.g 1) remove “Theoretical” or 2) Biophysical modelling of dopaminergic denervation landscapes in the striatum revel new therapeutic strategies etc.

5) In the abstract and the introduction, the authors mention that their theoretical work provides experimentally testable predictions, but do not specify them explicitly. For the clarity, I suggest spelling them out both in the abstract and/or at least in the introduction.

6) Further, the materials and methods sections refers to the results as it would have been placed after the results section (e.g. referring to “described algorithms”). Also, several abbreviations (e.g. CAC, RD, PLD, SID etc.) are not explained on the first appearance (while they are explained further in the results). Please, correct.

7) In the results, page 6, parameter Vm is not explicitly explained. The authors should also explain the reasoning behind choosing specific numerical values for the model’s parameters listed in Table S1 - and provide corresponding references if these parameters have been previously estimated experimentally and/or theoretically. Otherwise, it is not clear why, for example, the contribution of diffusion and degradation of dopamine to local dopamine concentration is stated to be small.

8) Explain abbreviations also in the figure legends - e.g. what in NC in Fig. 5?

9) In the first sentence of the last paragraph of the introduction (page 5) the correct text should presumably be “...we developed a series of biophysical models...”
