# Erratum: Heltberg et al., “Biophysical Modeling of Dopaminergic Denervation Landscapes in the Striatum Reveals New Therapeutic Strategy”

**DOI:** 10.1523/ENEURO.0147-22.2022

**Published:** 2022-04-25

**Authors:** Mathias L. Heltberg, Hussein N. Awada, Alessandra Lucchetti, Mogens H. Jensen, Jakob K. Dreyer, Rune N. Rasmussen

**Affiliations:** 1 Laboratoire de Physique, École Normale Supérieure, 75231 Paris Cedex 05, France; 2 Niels Bohr Institute, University of Copenhagen, 2100 Copenhagen, Denmark; 3 Section of Surgical Pathophysiology, University Hospital Copenhagen, 2200 Copenhagen, Denmark; 4 Department of Anesthesiology, University Hospital Copenhagen, 2200 Copenhagen, Denmark; 5 Department of Neuroscience, University of Copenhagen, 2200 Copenhagen, Denmark; 6 Department of Bioinformatics, H Lundbeck A/S, 2500 Valby, Denmark; 7 Center for Translational Neuromedicine, University of Copenhagen, 2200 Copenhagen, Denmark

In the article, “Biophysical Modeling of Dopaminergic Denervation Landscapes in the Striatum Reveals New Therapeutic Strategy,” by Mathias L. Heltberg, Hussein N. Awada, Alessandra Lucchetti, Mogens H. Jensen, Jakob K. Dreyer, and Rune N. Rasmussen, which was published online February 14, 2022, Mathias L. Heltberg’s and Hussein N. Awada’s affiliations were listed incorrectly. Mathias L. Heltberg’s affiliations are Laboratoire de Physique, École Normale Supérieure, 75231 Paris Cedex 05, France; and Niels Bohr Institute, University of Copenhagen, 2100 Copenhagen, Denmark. Hussein N. Awada’s affiliations are Section of Surgical Pathophysiology, University Hospital Copenhagen, 2200 Copenhagen, Denmark; and Department of Anesthesiology, University Hospital Copenhagen, 2200 Copenhagen, Denmark. The article has been corrected online, and the correct author affiliations appear below.

**Mathias L. Heltberg,^1,2^ Hussein N. Awada,^3,4^ Alessandra Lucchetti,^2^ Mogens H. Jensen,^2^ Jakob K. Dreyer,^5,6^ and Rune N. Rasmussen^7^**

^1^Laboratoire de Physique, École Normale Supérieure, 75231 Paris Cedex 05, France, ^2^Niels Bohr Institute, University of Copenhagen, 2100 Copenhagen, Denmark, ^3^Section of Surgical Pathophysiology, University Hospital Copenhagen, 2200 Copenhagen, Denmark, ^4^Department of Anesthesiology, University Hospital Copenhagen, 2200 Copenhagen, Denmark, ^5^Department of Neuroscience, University of Copenhagen, 2200 Copenhagen, Denmark, ^6^Department of Bioinformatics, H Lundbeck A/S, 2500 Valby, Denmark, and ^7^Center for Translational Neuromedicine, University of Copenhagen, 2200 Copenhagen, Denmark

