# Supplementary figures and images for: Biophysical Modeling of Dopaminergic Denervation Landscapes in the Striatum Reveals New Therapeutic Strategy
Source: eNeuro. 2022 Mar 2;9(2):ENEURO.0458-21.2022. doi: 10.1523/ENEURO.0458-21.2022 (PMC8896595; doi:10.1523/ENEURO.0458-21.2022)

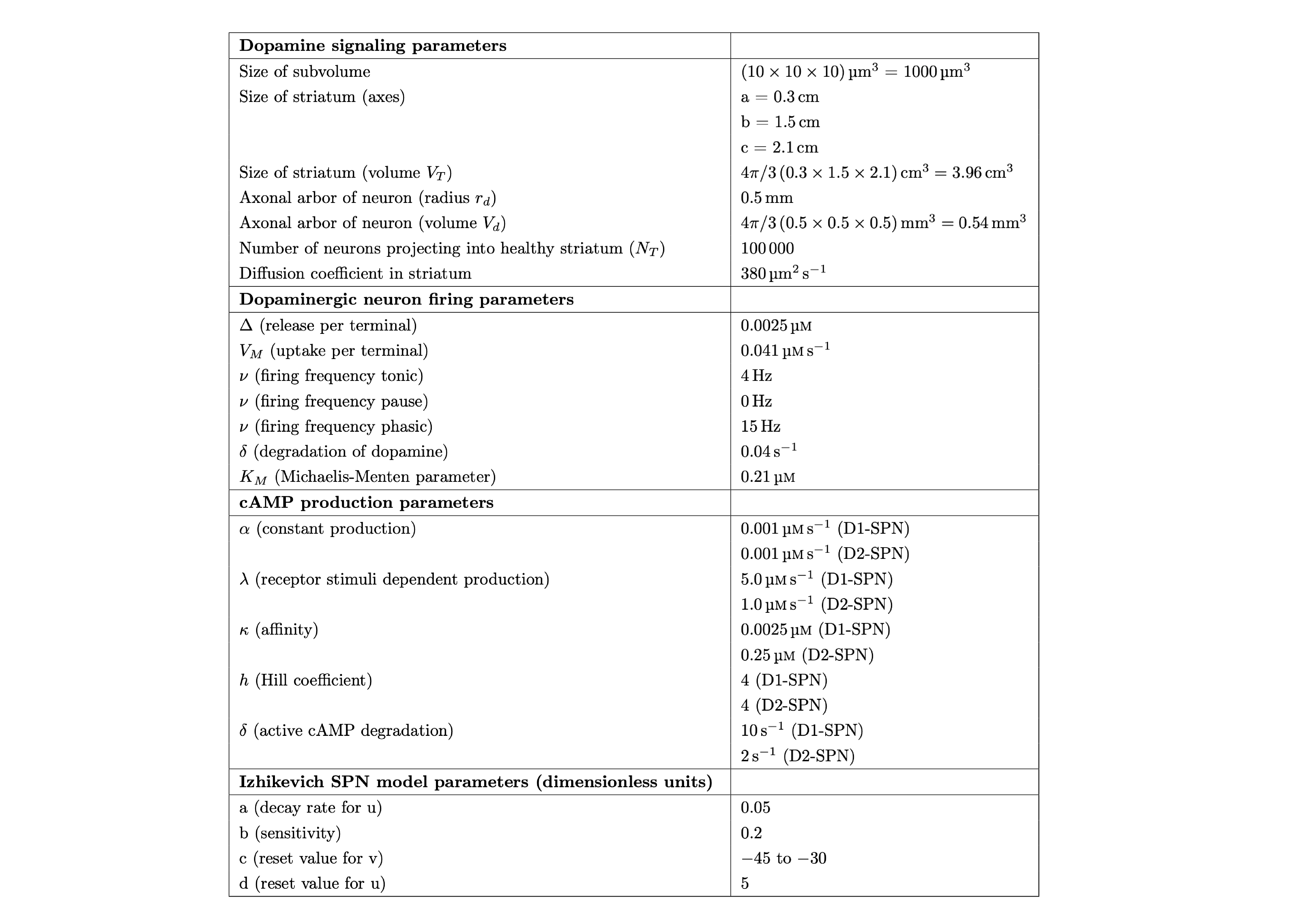

Supplement: Extended Data Figure 1-1 — Model parameters. Table summarizing the biophysical parameter values used for the respective computational models. Download Figure 1-1, TIF file. [file enu-eN-NWR-0458-21-s03.tif]

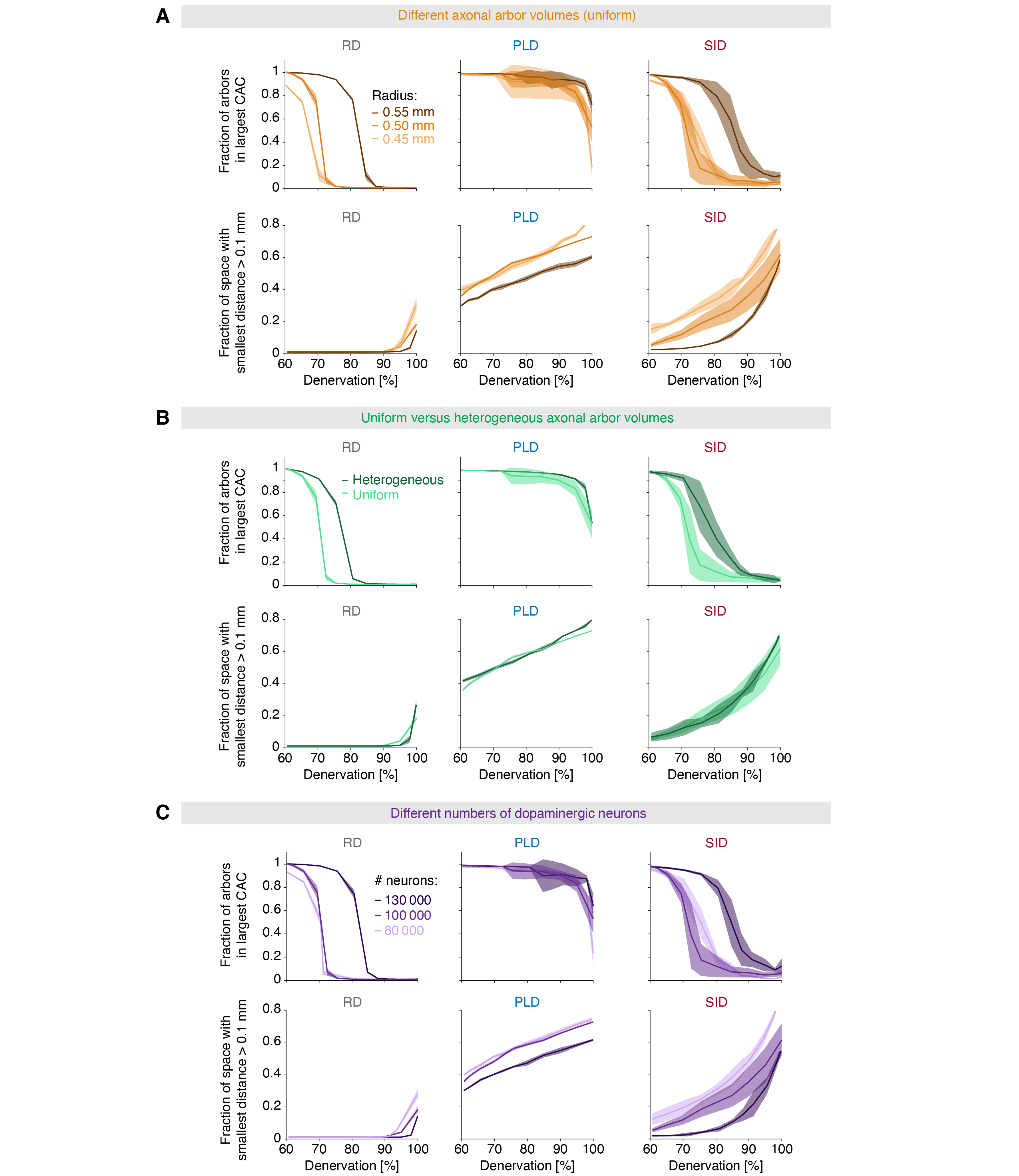

Supplement: Extended Data Figure 2-1 — Spatial denervation evolutions are robust to changes in key parameters. A, Effects of varying the volume of axonal arbors (radius: 0.45, 0.5, or 0.55 mm) uniformly across the arbor population in the three denervation models. Upper, Fraction of arbors belonging to the largest CAC as a function of denervation. Lower, Fraction of striatal space with smallest distance to nearest arbor larger than 0.1 mm (isolated area) as a function of denervation. B, Same as in A, but with the volume of arbors following a δ function (Vn=δ(Vn−V0)) versus heterogeneous distribution so the volume follows a normal distribution (Vn=V010×N(0,1)). For both cases, V0=43πr0 where r0 is the standard radius (r0=0.5mm). C, Same as in A, but for different numbers of dopaminergic neurons in the healthy state (80,000, 100,000, or 130,000 neurons). In A–C, full line is the mean, and shading is the SD. RD, random denervation; PLD, prion-like denervation; SID, stress-induced denervation; CAC, contiguous arbor class. Download Figure 2-1, TIF file. [file enu-eN-NWR-0458-21-s04.tif]

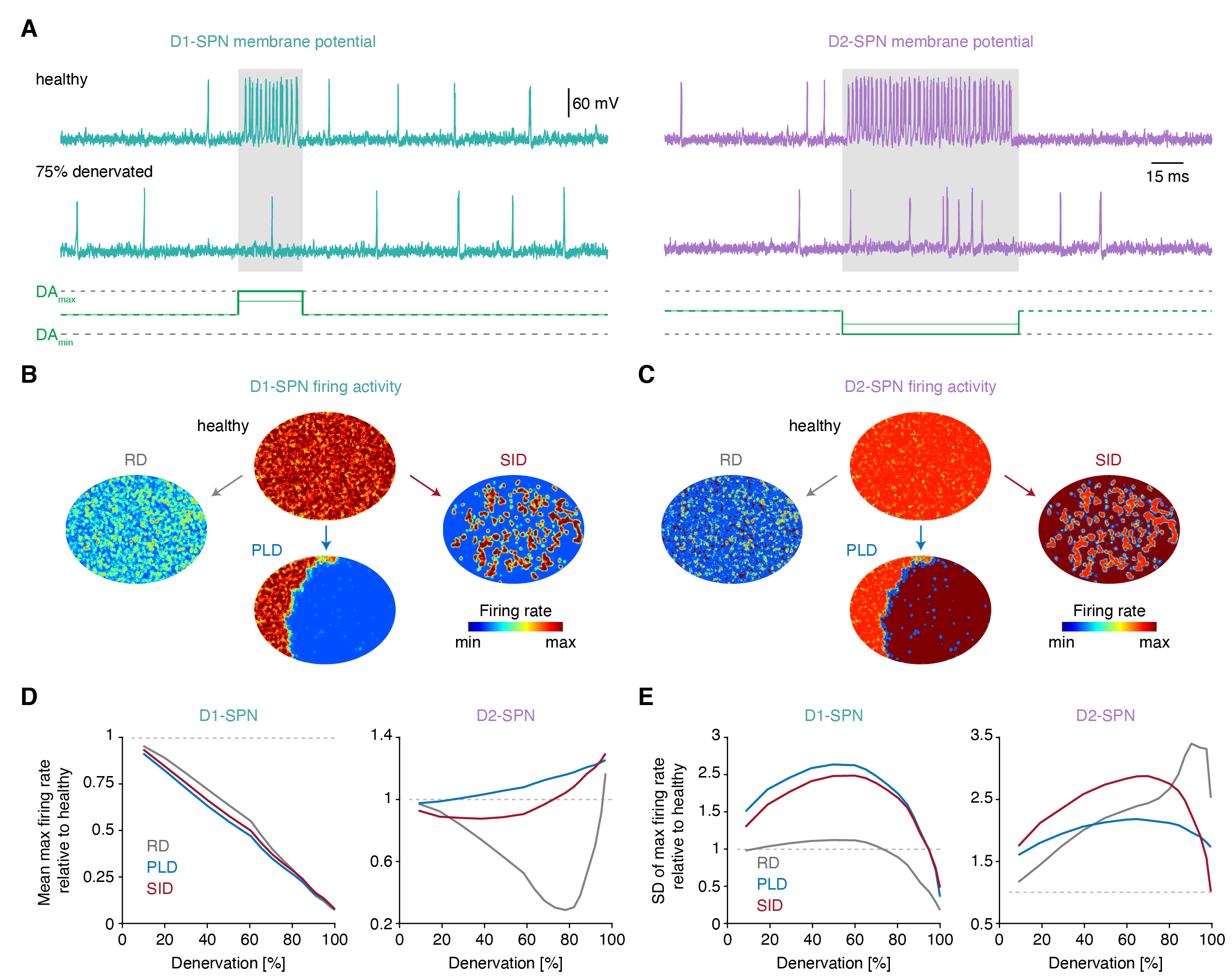

Supplement: Extended Data Figure 3-1 — Distinct denervation patterns differentially affect local and global striatal SPN firing activity in the Izhikevich model. A, Membrane potential of D1-SPN (left) and D2-SPN (right) in the healthy and 75% denervated striatum in response to DA signaling, modelled using the Izhikevich model. B, C, Maximal firing activity of D1- and D2-SPNs across space in the healthy and 75% denervated striatum for the three denervation patterns: RD, PLD, and SID. D, E, Spatial mean and SD of maximum firing activity in D1- and D2-SPNs as a function of denervation. SPN, spiny projection neuron; D1, D1-class dopamine receptor; D2, D2-class dopamine receptor; DA, dopamine. Download Figure 3-1, TIF file. [file enu-eN-NWR-0458-21-s05.tif]

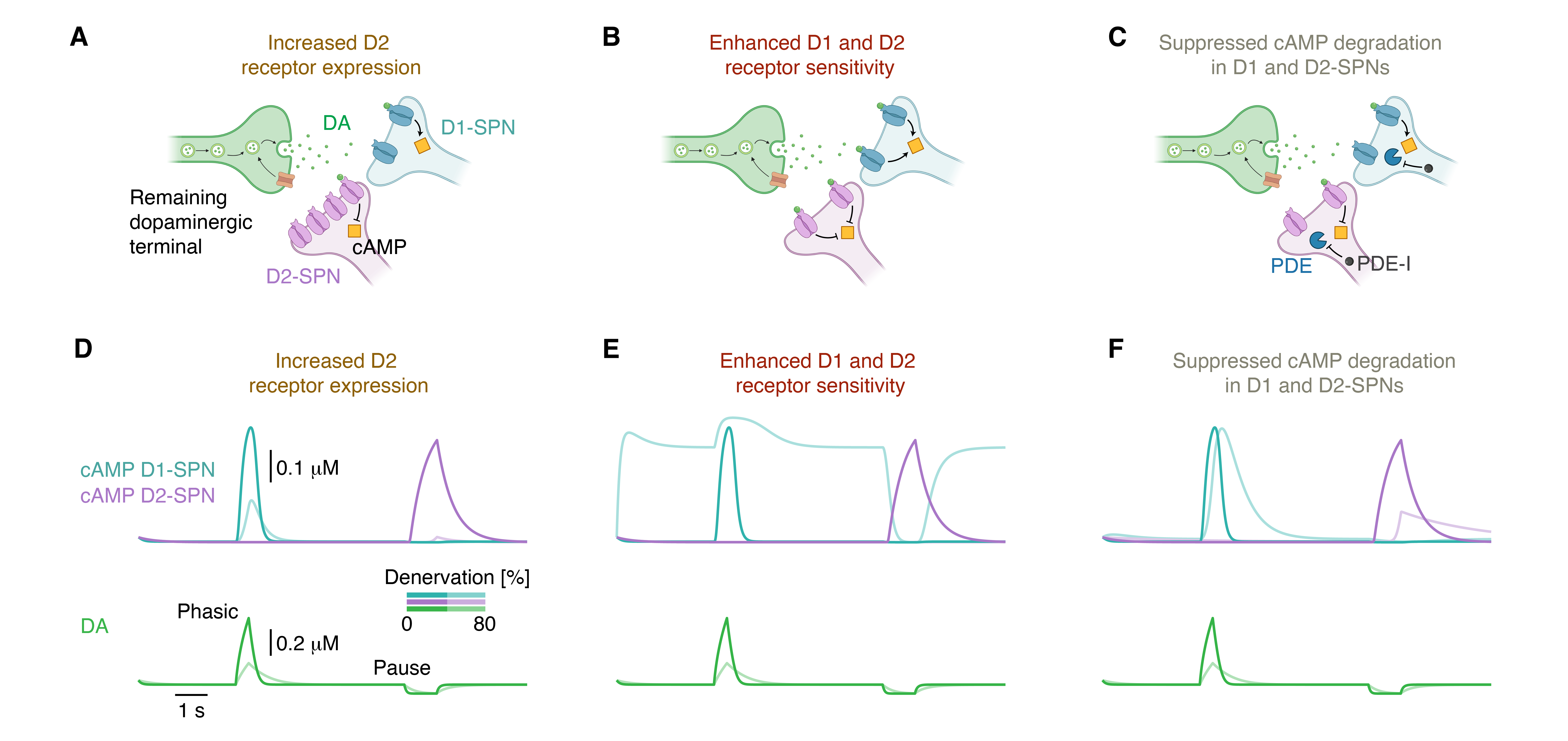

Supplement: Extended Data Figure 5-1 — Three distinct postsynaptic mechanisms fail to preserve DA signaling in the denervated striatum. A–C, Diagrams of the modelled postsynaptic compensatory mechanisms: increased D2 expression (A), enhanced D1 and D2 sensitivity (B), and suppressed cAMP degradation in D1- and D2-SPNs mediated by, for example, a PDE inhibitor (PDE-I; C). D–F, Example traces showing cAMP in D1- and D2-SPNs as a function of DA signaling at 0% and 80% denervation in the different postsynaptic compensation models. DA, dopamine; D2, D2-class dopamine receptor; D1, D1-class dopamine receptor; SPN, spiny projection neuron; PDE, phosphodiesterase. Download Figure 5-1, TIF file. [file enu-eN-NWR-0458-21-s06.tif]

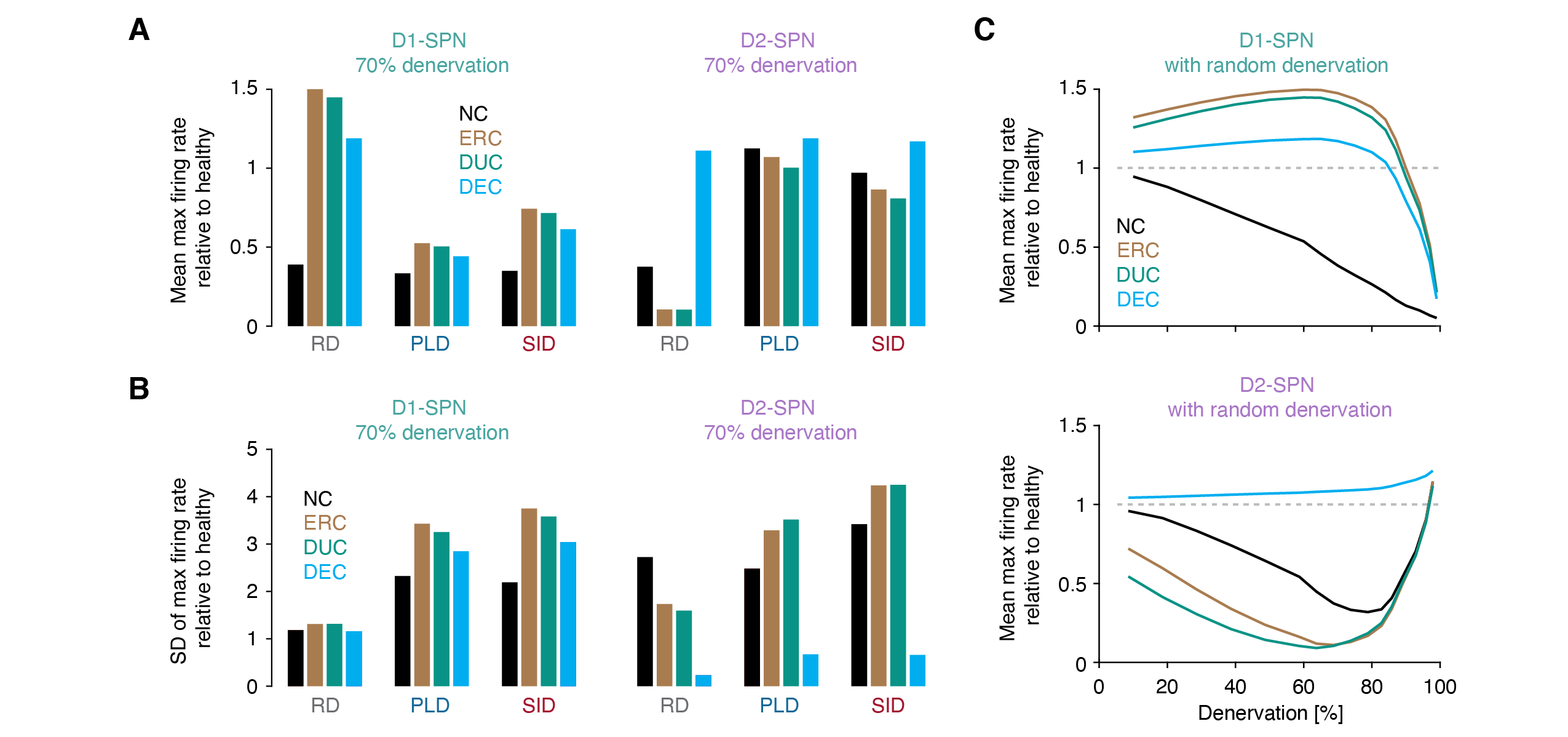

Supplement: Extended Data Figure 5-2 — A dual presynaptic compensation strategy preserves SPN firing activity in the Izhikevich model in spite of severe denervation. A, B, Spatial mean and SD of maximum firing activity in D1- and D2-SPNs as a function of denervation pattern (RD, PLD, and SID) and compensation model (ERC, DUC, and DEC). C, Spatial mean of maximum firing activity in D1- and D2-SPNs as a function of denervation and compensation model in the randomly denervated striatum. SPN, spiny projection neuron; D1, D1-class dopamine receptor; D2, D2-class dopamine receptor; RD, random denervation; PLD, prion-like denervation; SID, stress-induced denervation; ERC, enhanced release compensation; DUC, decreased uptake compensation; DEC, dual enhanced compensation. Download Figure 5-2, TIF file. [file enu-eN-NWR-0458-21-s07.tif]
